# Surgical Apgar Score can accurately predict the severity of post-operative complications following emergency laparotomy

**DOI:** 10.1186/s12893-023-02088-2

**Published:** 2023-07-06

**Authors:** Victor Meza Kyaruzi, Douglas E. Chamshama, Ramadhani H. Khamisi, Larry O. Akoko

**Affiliations:** 1 Department of General Surgery, Muhimbili University of Health and Allied Science, Dar Es Salaam, Tanzania; 2 Department of General Surgery, Muhimbili National Hospital, Dar Es Salaam, Tanzania

**Keywords:** Surgical apgar score (SAS), Complication severity, Comprehensive Complication Index (CCI), Predictive accuracy

## Abstract

**Background:**

The Surgical Apgar Score (SAS) describes a feasible and objective tool for predicting surgical outcomes. However, the accuracy of the score and its correlation with the complication severity has not been well established in many grounds of low resource settings.

**Objective:**

To determine the accuracy of Surgical Apgar Score in predicting the severity of post-operative complications among patients undergoing emergency laparotomy at Muhimbili National Hospital.

**Methods:**

A prospective cohort study was conducted for a period of 12 months; patients were followed for 30 days, the risk of complication was classified using the Surgical Apgar Score (SAS), severity of complication was estimated using the Clavien Dindo Classification (CDC) grading scheme and Comprehensive Complication Index (CCI). Spearman correlation and simple linear regression statistic models were applied to establish the relationship between Surgical Apgar Score (SAS) and Comprehensive Complication Index (CCI). The Accuracy of SAS was evaluated by determining its discriminatory capacity on Receiver Operating Characteristics (ROC) curve, data normality was tested by Shapiro–Wilk statistic 0.929 (*p* < 0.001).Analysis was done using International Business Machine Statistical Product and Service Solution (IBM SPSS) version 27.

**Results:**

Out of the 111 patients who underwent emergency laparotomy, 71 (64%) were Male and the median age (IQR) was 49 (36, 59).The mean SAS was 4.86 (± 1.29) and the median CCI (IQR) was 36.20 (26.2, 42.40). Patients in the high-risk SAS group (0–4) were more likely to experience severe and life-threatening complications, with a mean CCI of 53.3 (95% CI: 47.2–63.4), compared to the low-risk SAS group (7–10) with a mean CCI of 21.0 (95% CI: 5.3–36.2). A negative correlation was observed between SAS and CCI, with a Spearman r of -0.575 (*p* < 0.001) and a regression coefficient b of -11.5 (*p* < 0.001). The SAS demonstrated good accuracy in predicting post-operative complications, with an area under the curve of 0.712 (95% CI: 0.523–0.902, *p* < 0.001) on the ROC.

**Conclusion:**

This study has demonstrated that SAS can accurately predict the occurrence of complications following emergency laparotomy at Muhimbili National Hospital.

## Introduction

Post-operative morbidity and mortality reduction is the fundamental goal of every surgical procedure [1]. The key to reduce post-operative morbidity and mortality is to employ effective perioperative management of patients with objective and scrupulous evaluation. Emergency laparotomy is one of the most performed delicate surgical procedure usually done in patients who already have sustained severe physiological stress and hemodynamic instability antecedent to severe hemorrhage, electrolyte imbalance, systemic inflammatory response and sepsis [2–5]. Based on these factors patients are usually susceptible to increased risk of developing detrimental complications and high rate of mortality within 30 days of post-operative period. Post-operative respiratory infection rate accounts for about 40% of patients who undergo abdominal surgery [6, 7].

Risk scoring system is the best modality approach as it provides a standard means of quantifying the patient’s risk for developing complications based on several factors including the morbidity status of patient [8].

However, vast majority of risk-scoring systems are not feasibly calculated at the bedside; due to their numerous demands for estimation which include laboratory investigations inasmuch as also clinicians and surgeons do not regularly apply them for patients assessment and stratification [9].

Surgical Apgar Score (SAS) describes a feasible, immediate and an objective modality of determining surgical outcomes [10]. It is a ten point score, which applies three haemodynamic parameters, the lowest heart rate, the lowest mean arterial pressure and estimated blood loss during surgery to predict the attributable complication risk after general or vascular surgery [11].

Among the most contemporary risk tools available for surgical patients, SAS could provide a feasible instrument for its routine utility and applicability, it would emanate an immediate and objective evaluation means guiding the surgical team to estimate the risk of post-operative complication upon completion of operation using the simple haemodynamic variables. SAS can be used to stratify patients into three categories of risk levels classified as low risk, medium risk and high risk. Patients in high-risk group (SAS 0- 4) are 16 folds more likely to experience major complications than patients in low risk (SAS 7–10) [11].

The accuracy of SAS have never been tested at our setting and its predictive accuracy for severity of postoperative complications is still uncertain since various studies have not yet proven the correlation between the stratified risk levels of SAS and the severity of the complications [12, 13]. It has been observed that there is increased rate for occurrence of major complications and high mortality among patients with the lowest SAS ≤ 4, however there is a paucity of evidence to justify if any statistical correlation exists between the risk level of SAS and severity of post-operative complication [11, 14].

## Material and methods

A prospective cohort study was conducted in Department of General Surgery at Muhimbili National Hospital. The study aimed to determine the accuracy of Surgical Apgar Score for prediction of post-operative complication severity among patients who underwent emergency laparotomy at Muhimbili National Hospital. We also aimed to describe the severity pattern of post-operative complication among patients who underwent emergency laparotomy and to evaluate the correlation between SAS and the severity of post-operative complication following emergency laparotomy.

### Study area

Muhimbili National Hospital is a National Referral Hospital, Research Center and University teaching Hospital with 1,500-bed capacity, attending 2,000 outpatients per day and admitting 1,000 to 1,200 inpatients per week. The Department of Surgery is one among six departments of the Directorate of Surgical Services, this department has 120-bed capacity, it comprises of operating surgical ICU and two emergency Operating theatre rooms, imaging services and established Interventional Radiology unit. It is divided into five units according to their areas of specialization including Gastroenterology and General Surgery, Thoracic and General Surgery, Urology, Paediatric Surgery and Plastic and Reconstructive Surgery Unit.

### Sample estimation and selection

The total sample size of 112 participants was estimated using the Cochran formula to enable the prediction accuracy of the model at a power (1-β) of 80%, type 1 error of less than 5% and 95% confidence interval.

Using the Cochran formula:

$$n=\frac{\mathrm Z^2\;\times\;\mathrm p(1-\mathrm p)}{d^2}$$

Where

z = Score at 95% confidence interval (1.96)

p = 30 day mortality in patients undergoing emergency laparotomy (7.9%) [12]

d = margin of error (0.05%)

n = 112

During the study period we were able to recruit 111 patients who underwent emergency laparotomy from March 2021 to February 2022 by non-probability convenient sampling, patients with age above 18 years and ASA ≥ II were included while those who had severe anaemia Hb ≤ 7 g/dl and Sickle Cell Anaemia were excluded from the study.

### Data collection and measurements

The data were recorded on structured checklist. The data were collected from patients’ file and anesthesia logs. The structured checklist was developed incorporating the sociodemographic characteristics, clinical characteristics, variables for complication severity grading including the Clavien Dindo Classification domains (CDC) and Comprehensive Complication Index (CCI), Surgical Apgar Score Parameters. The checklist was pretested for validity and reliability using the internal consistency. The cronbach’s alpha threshold was more than 0.7 and we accepted and adopted the tool for data collection purpose.

Surgical Apgar Score (SAS) for patients was estimated by computing three inraoperative haemodynamic parameters the lowest mean heart rate (HR), estimated blood loss (EBL) and the lowest mean arterial pressure (MAP), SAS was further stratified into three levels of risk as high risk (SAS 0–4), moderate risk (SAS 5–6) and low risk (SAS 7–10), Table 1. Blood loss was estimated using the Gross formula [15]. Patients were followed within a period of 30 days after surgery, the observed complications were recorded and the severity of complication was graded according to modified Clavien Dindo Classification scheme [16–18]. The grades of severity were quantified numerically using the Weighted Comprehensive Complication Index (CCI) calculator [19–21].

**Table 1:** The ten-point surgical Apgar score

Parameter	Points				
	0	1	2	3	4
Estimated Blood loss (ml)	> 1000	601–1000	101–600	≤ 100	-
Lowest heart rate (beats/min)	> 85	76–85	66–75	56–65	≤ 55
Lowest MAP (mmHg)	< 40	40–54	55–69	≥ 70	-

*MAP *Mean Arterial Pressure

The Gross formula for estimation of blood loss is described below.

$$\mathrm{EBL}=\mathrm E\mathrm B\mathrm V\;\times\;\lbrack(\frac{\mathrm{HBi}-\mathrm{HBf}}{\mathrm{HBi}+\mathrm{HBf}})\rbrack/2+(500\;\times\;\mathrm{Tu})$$

Where,

EBV = Estimated blood volume (body weight in kgs × 70 ml/kg)

HBi = Pre-operative hemoglobin (g/dl),

HBf = Post-operative hemoglobin (g/dl) around 24 h after surgery

Tu = Sum of units of blood transfused (i.e. whole blood, packed red blood cell transfused).

The numerical value of complication severity for every individual patient was calculated using the weighted Comprehensive Complication Index (CCI) Calculator. The correlation for complication severity expressed in CCI and the SAS risk level was tested using the Spearman rank (r) and simple linear regression analysis. The predictive accuracy of SAS for post-operative complication was tested by considering its discrimination capacity by Area under Curve on ROC. The analysis was conducted using the International Business Machine Statistical Product and Service Solution (IBM SPSS) version 27.

### Ethical clearance

The ethical clearance approval protocol for this study was obtained from Institutional Review Board of Muhimbili University of Health and Allied Sciences (IRB-MUHAS), REF DA.282/298/01.C.

## Results

Among patients who were recruited the Male were predominant at 71 (64.0%), with a median age of 49 (36, 59) most of the patients were in the age group of between 61 and 75 years in 42 (37.8%) followed by those between 44 and 60 years in 26(23.4%). The mean body weight in Kg and the mean baseline haemoglobin in g/dl of patients in were 63.8 ± 9.93 and 11.22 ± 2.98. Peritonitis and intestinal obstruction were the leading indications for emergency laparotomy with proportion of 44.1% and 36.9% respectively Table 2.

**Table 2:** Baseline characteristics and indications for surgery among patients who underwent emergency laparotomy at Muhimbili Nationa Hospital 2021

Variables	Mean ± SD	Frequency (%)
**Age group**		
< 44		19 (17.11)
44–60		26 (23.40)
61–75		42 (37.80)
> 75		24 (21.60)
**Median age**	49 (36,59)	
**Sex**		
Male		71 (64.00)
Female		40 (36.00)
**Mean body weight (Kgs)**	63.80 (± 9.93)	
**Mean Haemoglobin (g/dl)**	11.22 (± 2.98)	
**Pre-operative diagnosis**		
Peritonitis		49 (44.10)
Intestinal obstruction		41 (36.90)
Abdominal visceral injury		7 (6.30)
Diaphragmatic tear		2 (1.80)
Enterocutaneous fistula		6 (5.40)
Others		6 (5.40)
**TOTAL**		**(** ***n*** ** = 111)**

### Perioperative clinical characteristics of the patients

The mean SAS for patients was 4.86 (± 1.29) and the median CCI for a 30-day complication was 36.20 (26.20, 42.40) with mortality rate of 16.2%, 86.5% of complications occurred within ≤ 10 days, Tables 3 and 4.

**Table 3:** SAS variables of patients who underwent emergency laparotomy at MNH in 2021

Variables	Mean ± SD	Frequency (%)
**Median estimated blood loss ( IQR)**	747.00 (361.00, 1085.00)	
**Mean hemoglobin (g/dl)**	10.60 (± 8.57)	
**Mean lowest DBP (mmHg)**	55.42 (± 12.79)	
**Mean lowest SBP (mmHg)**	94.05 (± 18.10)	
**Mean lowest heart rate (bpm)**	87.95 (± 16.66)	
**Mean lowest MAP (mmHg)**	67.69 (± 13.45)	
**Median CCI (IQR)**	36.20 ( 26.20, 42.40)	
**Mean SAS**	4.86 (± 1.29)	
**Complication onset (days)**		
0–11		96 ( 86.50)
11- 20		13 (11.70)
21–30		2 (1.80)
**Complication Severity (CDC)**		
Low grade ( I-II)		9 ( 8.10)
High grade (III-V)		102 (91.90)
**TOTAL**		**(** ***n*** ** = 111)**

**Table 4:** The accuracy and precision values for Surgical Apgar Score

Cutpoint	Sensitivity (%)	Specificity (%)	PPV (%)	NPV (%)	Youden’s index	AUC	Metric Score
5	61.76%	77.78%	96.92%	15.22%	0.395	0.712	1.40

### Severity pattern for a 30-day post operative complication

**Fig. 1:**
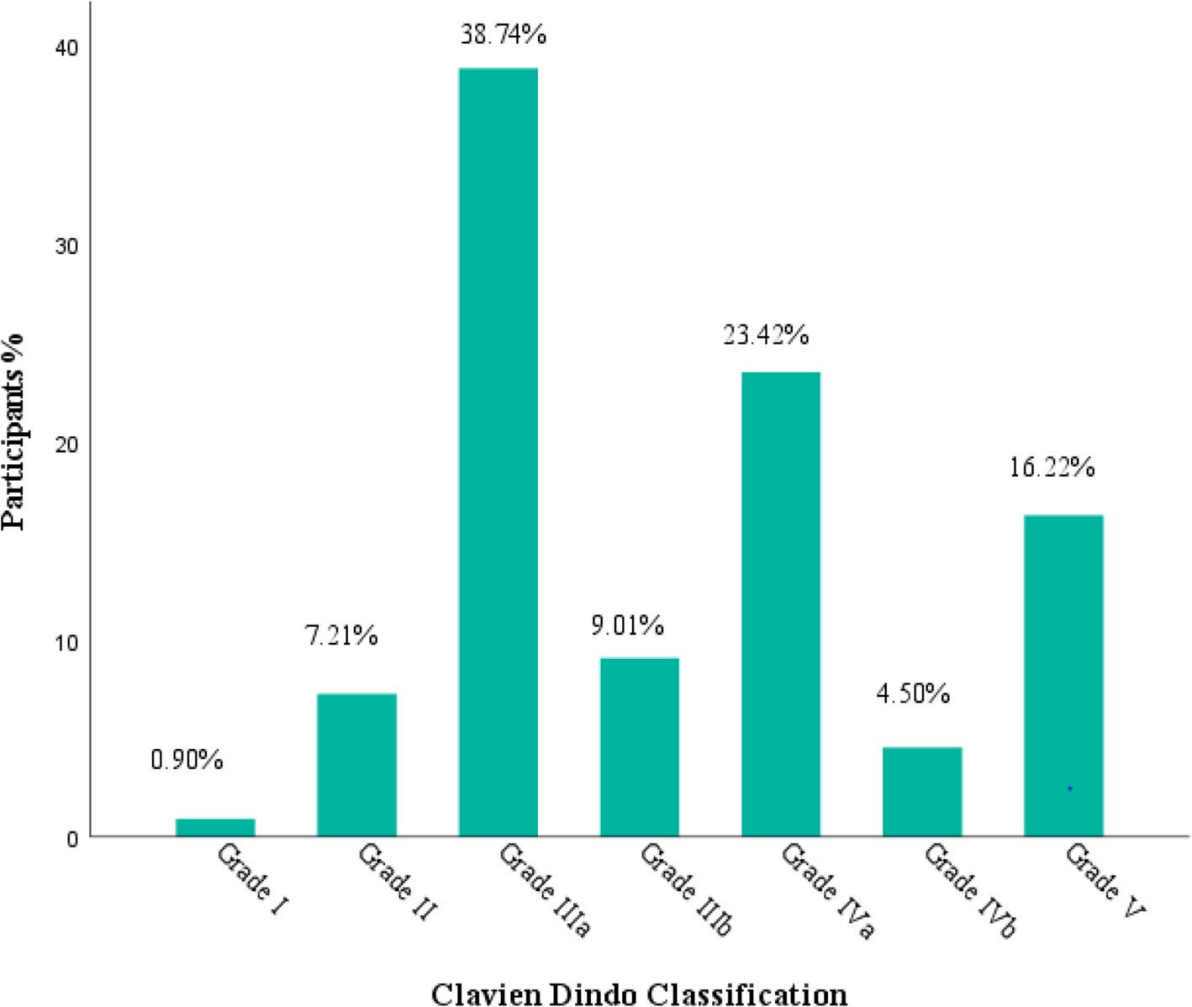
Clavien-Dindo classification of complications among post emergency laparotomy patients at MNH 2021

Complications were graded based on modified Clavien Dindo Classification scheme, category I-II and III-V were classified as Low and High grade respectively. Most of severe and detrimental complications were attributable to high grade with a preponderance score of grade III-V, Fig. 1.

**Fig. 2:**
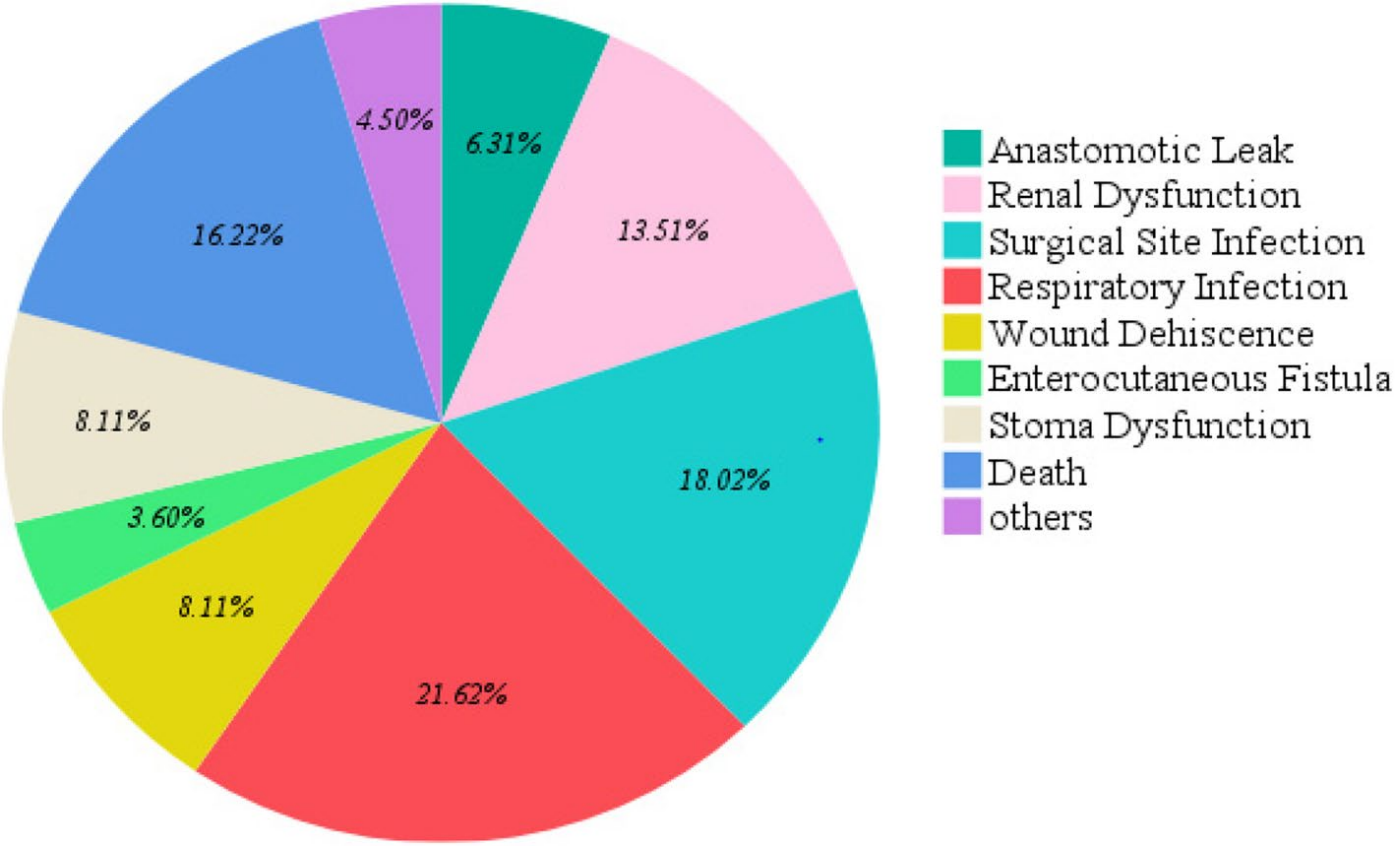
Pie chart showing complications occurring among emergency laparotomy patients at MNH in 2021

Several complications were identified within 30 days of post operative period and each individual patient developed at least one or more complication, respiratory infection was the most prevalent 21.62% followed by death 16.22% as illustrated in Fig. 2.

**Fig. 3:**
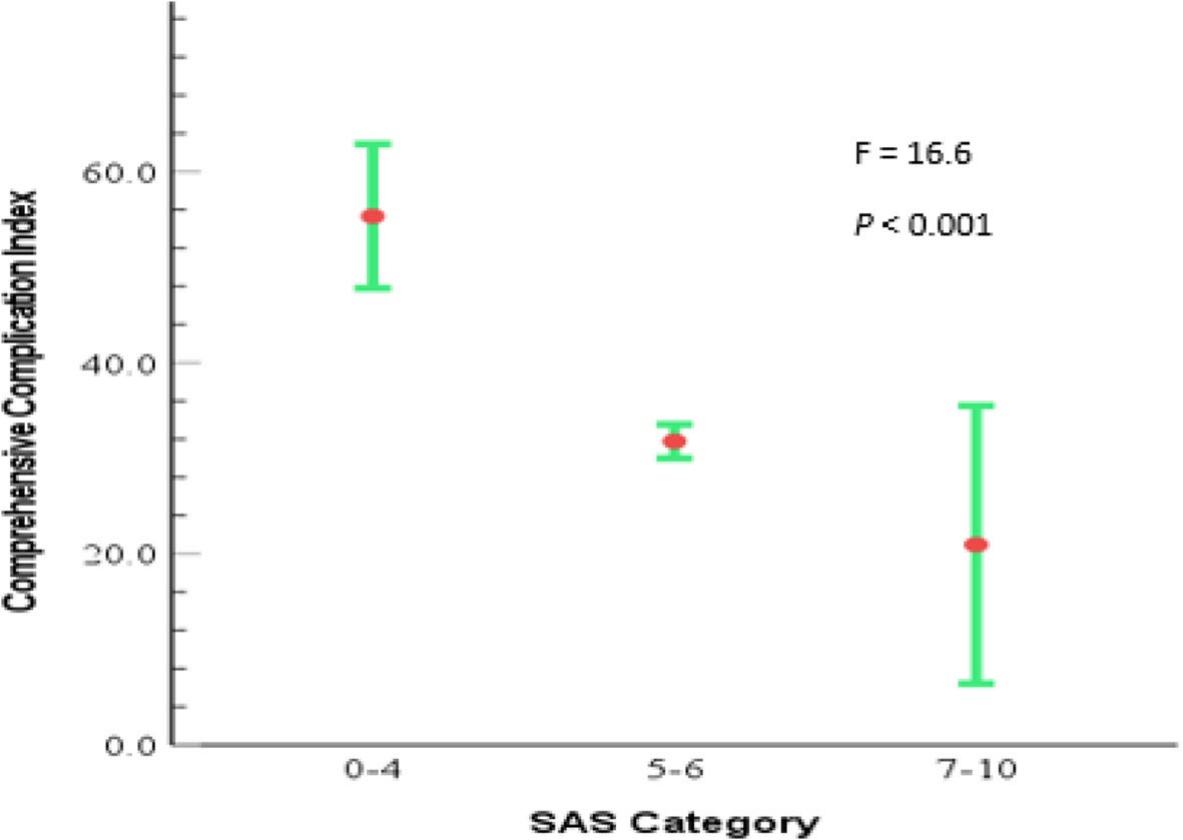
Mean CCI according to SAS category

Patients were stratified into three categories of risk level as high, medium and low based on the SAS classification 0–4, 5–6 and 7–10 respectively. It was observed that patients in high-risk group were more likely to develop severe and life threatening complications with the mean CCI of 53.3 (47. 2- 63.4, 95% CI). The medium risk group had a mean CCI of 31.8 (28.9–35.7, 95% CI) and the low risk group was less likely to develop severe complication with the mean CCI of 21.0 (5.3–36.2, 95% CI), One Way ANOVA was done to compare the means which yielded the significant mean difference (F = 16.6,* p* < 0.001) Fig. 3.

### The correlation of comprehensive complication index and surgical Apgar score

**Fig. 4:**
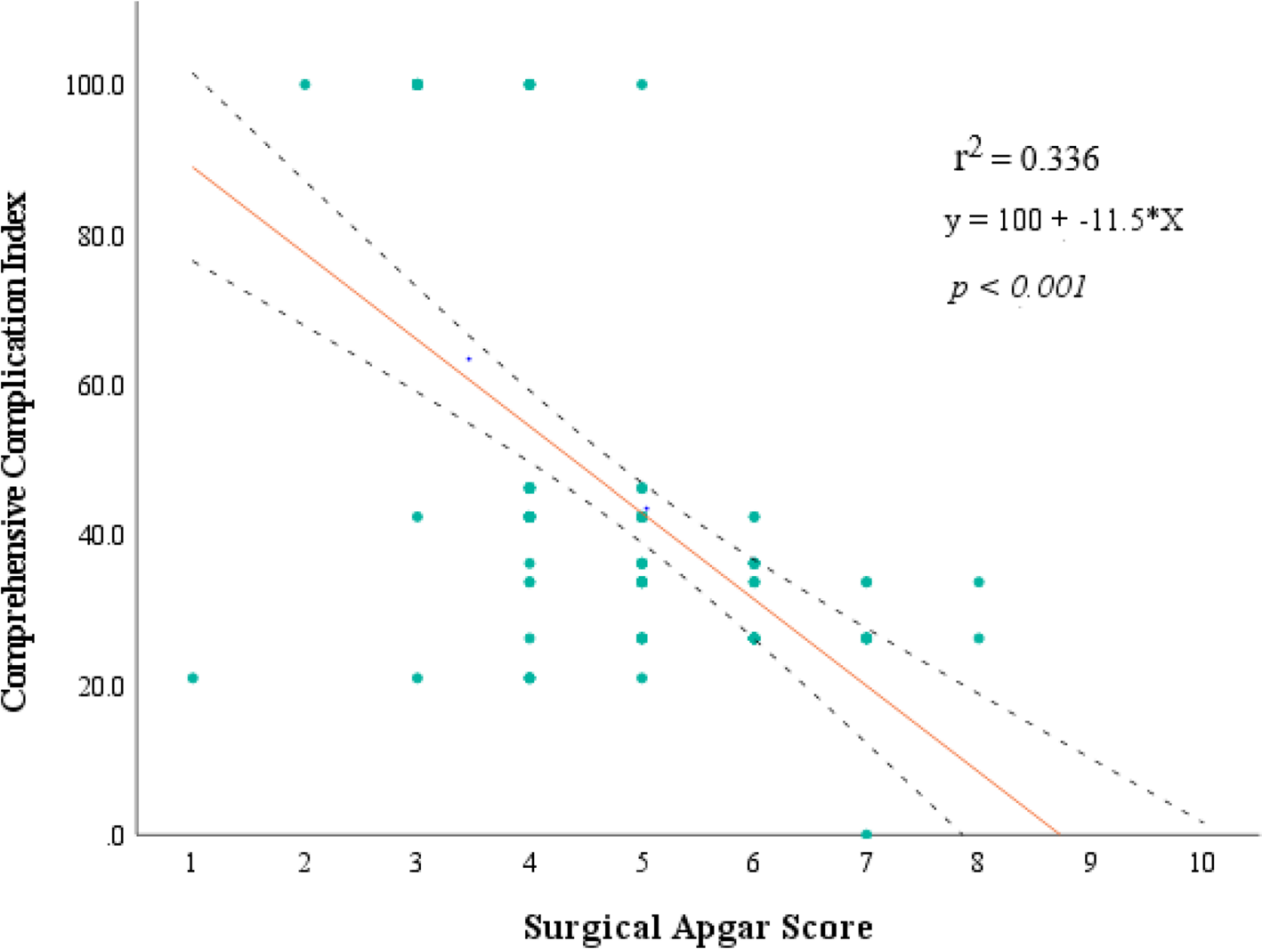
CCI and SAS regression scatter plot

The correlation test was performed to determine if there is any existing relationship between Surgical Apgar score and the complication severity expressed in CCI. The spearman coefficient shows an existing negative weak correlation, *r* = -0.575, *p* < 0.001. A simple linear regression analysis was also performed to test if there is any existing degree of dependence for Comprehensive Complication Index by changes of value in Surgical Apgar Score. The statistic model y = 100 + -11.5*X was derived and revealed a negative relationship between the two variables with regression coefficient, b = -11.5, *p* < 0.001, which depicts a decrease change of CCI by 11.5 for every increment of one value of SAS with coefficient of determination, r^2^ = 0.336, *p* < 0.001 Fig. 4.

### The accuracy of SAS for prediction of post-operative complication severity

**Fig. 5:**
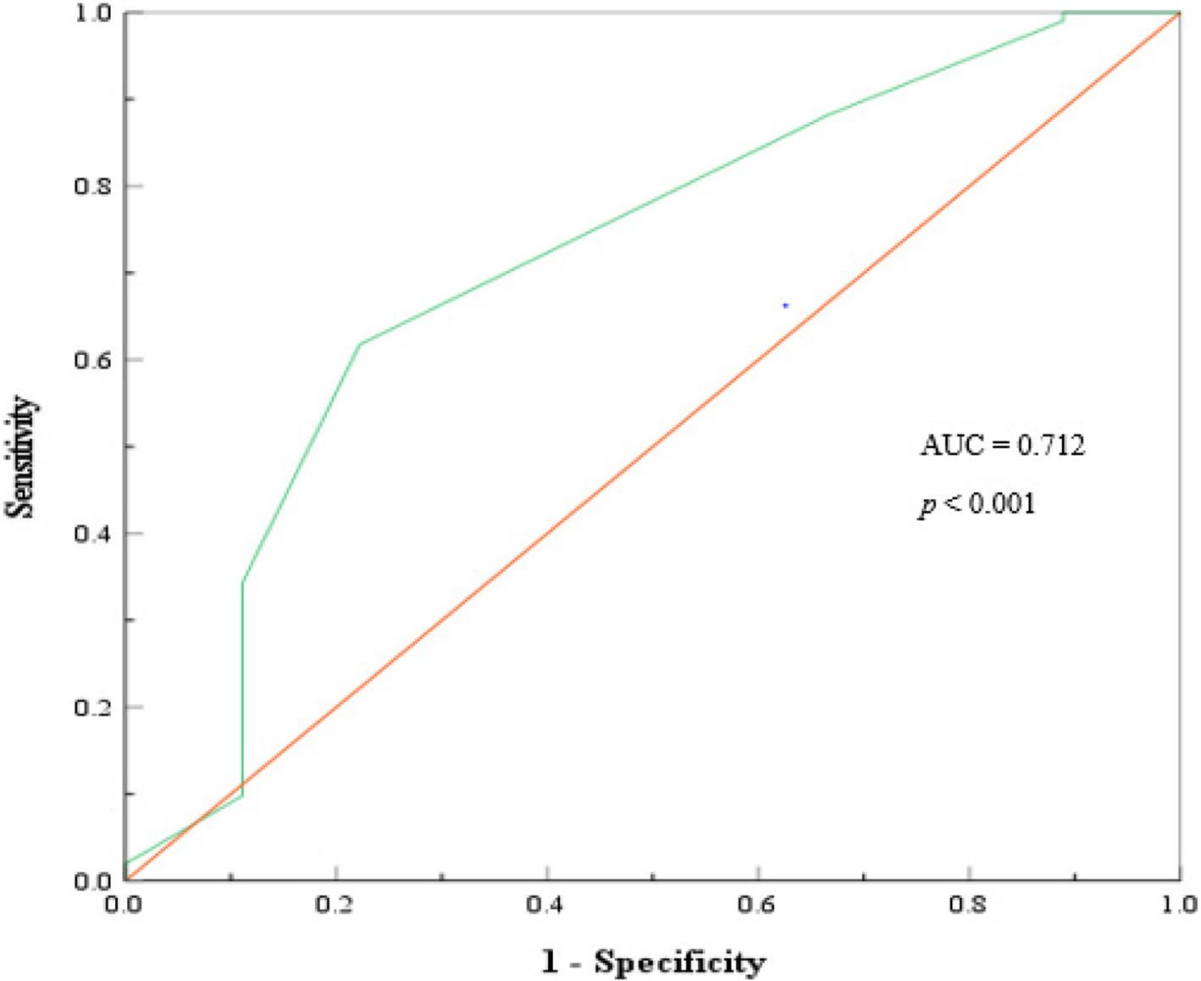
Receiver operating characteristic curve showing the area under curve for complication predictive accuracy of SAS

It was observed that within the period of 30 days, every individual patient developed at least one complication, and each complication was classified into a dichotomous variable as either low or high grade outcome based on modified Clavien Dindo. The discriminatory capacity of SAS was determined using the ROC and Area Under Curve was 0.712 (0.523–0.902, 95% CI, *p* < 0.001)**,** the AUC indicates that SAS is satisfactory score for prediction of postoperative complication severity (0.7 < AUC < 0.8) as shown in Fig. 5. Sensitivity 61.76%, Specificity 77.78%, PPV 96.92% and NPV 15.22%, *p* < 0.035 Tables 3 and 4 and diagnostic cutoff point of 5 for SAS was estimated Table 5.

**Table 5:** Classification of participants based on cut-off of 5 Surgical Apgar Score

Surgical Apgar Score	Clavien Dindo Classification (CDC)	
	**High grade**	**Low grade**
< 5	63 (TP)	2 (FP)
≥ 5	39 (FN)	7 (TN)
Total	102	9

## Discussion

The study aimed at determining the accuracy of SAS for predicting the severity of post-operative complications among patients undergoing emergency laparotomy. Our study revealed that the mean Surgical Score (SAS) at Muhimbili National Hospital is 4.86 with the median Comprehensive Complication Index (CCI) of 36.20 (26.20, 42.40). Patients with the lowest SAS score (0–4), had the increased risk of developing severe and life threatening complication with high comprehensive complication index (CCI) score of 53.30.

In this study, it was determined that the most prevalent major complication was respiratory infection, 21.62% which accounted for increased morbidity and mortality, this related to what another study in India where it was reported an increased pulmonary morbidity accounting 34% with high rate of pneumonia 19.7% [22]. The increased rate of respiratory infections in our settings we postulated that it could have been influenced by the COVID19 pandemic reflected on the study period. The surgical site infection rate was 18.02%, this was more less compared to the overall surgical site infection rate of 25% which was described in the study done by Dullo et al. [12]. The mortality rate was 16.22% this was significantly higher than 7.9% mortality rate determined at Kitui District Hospital in Kenya [12], however it related with the results from other studies with mortality rate from 15–27.7% [13, 22].

A large proportion of patients in this study were scaled according to their complication severity as high grade (III-V) based on CDC scheme, this corresponded with the median Comprehensive Complication Index of 36.20 (26.2, 42.40). The mean SAS was 4.86 equivalent to the mean SAS derived from other studies with a mean range of 4–6 [12, 13].

SAS was stratified into three categories of risk level as High risk (0–4), Medium risk (5–6) and Low risk (7–10) [13, 23], there was increased degree of complication severity observed among patients with high risk, SAS ≤ 4, mean CCI 53.3 compared to the group with low risk,SAS ≥ 7 which had a mean CCI 21.0 this relationship was in keeping with findings from other studies tested by different statistic of associations [12, 13, 24, 25].

Using the linear regression analysis model we detected a significant negative correlation between SAS and the degree of complication severity estimated by weighted CCI. The spearman correlation coefficient was (*r* = -575,* p* = 0.001) and regression coefficient was (b = -11.5, *p* = 0.001), this implies that for every reduced one score of SAS it increases the risk of developing severe complications following emergency laparotomy by 11.5 folds.

Our study has revealed SAS has a good predictive accuracy with AUC 0.712 on Receiver Operating Characteristics (ROC), this indicates that SAS is a feasible tool and it can detect the difference between the severe versus non severe complications according to Clavien Dindo Classification (CDC) and CCI. It has produce the similar findings with other studies which have reported equivalent findings within the range of AUC 0.710 – 0.751.[13, 26, 27]. Moreover, other studies has proven a more optimum discriminatory power in the range of 0.75–0.796 of this tool [12, 13].

Several algorithms have been developed and used for risk stratification such as the American Society of Anesthesiologists Physical Status Classification System (ASA classification), the Physiologic and Operative Severity Score for enumeration of Mortality and morbidity (POSSUM), the Acute Physiology and Chronic Health Evaluation (APACHE), the Simplified Acute Physiology Score (SAPS) and American College of Surgeon NSQIP [28–31]. However, each of these systems has limitations and restricted applications. Although the ASA classification has proved to be a predictive preoperative risk factor in mortality models, its subjective nature and inconsistent scoring between providers make it less than ideal for performing evidence-based postoperative risk calculation [32, 33].

SAS has proven a feasible and objective instrument for identifying patients at high risk and has accurately demonstrated the good discriminatory capacity for prediction of post -operative complications severity following emergency laparotomy. The simplicity of SAS demystifies its empirical relevance for its utility and applicability in the resource constrained settings. The power and the strength of the Surgical Apgar Score include the capacity to compute the score quickly and objectively. Ultimately, the score may also prove useful in guiding preventive strategies such as optimizing intraoperative heart rate, arterial blood pressure and timely blood transfusion [34].

### Strengths and limitations

The outstanding strengths of this study encompass the use of simple haemodynamic measurements and nomograms to estimate the semiological endpoints of patients subjected to emergency laparotomy. However, it is not without limitations that a single center study nature, small sample size and convenient sampling rendering to selection bias altogether impose a deterrence for generalizability of our study.

## Conclusion

The high rate of morbidity and mortality associated with emergency laparotomy in our setting highlights the urgent need for an objective tool to determine risk levels and proactively optimize patient outcomes. Our study shows that the SAS is an accurate predictor of post-operative complications severity following emergency laparotomy at Muhimbili National Hospital.

## Data Availability

All materials pertinent to this research are available and shall be provided by the corresponding author upon reasonable request.

## Appendix

CDC Grades and the weighted CCI value

**Table:**

Grade	Definition	Single Value CCI
I	Any deviation from the normal postoperative course without the need for pharmacological treatment or surgical, endoscopic, and radiological interventions Allowed therapeutic regimens are drugs as antiemetics, antipyretics, analgesics, diuretics, electrolytes, and physiotherapy. This grade also includes wound infections opened at the bedside	8.7
II	Requiring pharmacological treatment with drugs other than such allowed for grade I complications Blood transfusions and total parenteral nutrition are also included	20.9
IIIa	Intervention not under general anesthesia	26.2
IIIb	Intervention under general anesthesia	33.7
IVa	Single organ dysfunction (including dialysis)	42.4
IVb	Multiorgan dysfunction (MOD)	46.2
V	Death of a patient	100
